# The potential effect of whey protein supplementation in post-bariatric surgery patients: a systematic review and meta-analysis of randomized controlled trials

**DOI:** 10.3389/fnut.2026.1832335

**Published:** 2026-06-23

**Authors:** Xuehan Zhao, Tianhang Shi, Xiaojuan Xie, Li Zhao, Tao Guo

**Affiliations:** 1 Department of Pathophysiology, School of Basic Medical Sciences, Shandong Second Medical University, Weifang, China; 2 Department of Hepatobiliary and Pancreatic Surgery, Affiliated Hospital of Shandong Second Medical University, School of Clinical Medicine, Shandong Second Medical University, Weifang, China; 3 Department of Clinical Microbiology, School of Laboratory Medicine, Shandong Second Medical University, Weifang, China

**Keywords:** anthropometry, bariatric surgery, body composition, lipid profiles, meta-analysis, randomized controlled trial, whey protein

## Abstract

**Background:**

Bariatric surgery is an effective intervention for severe obesity, and postoperative nutritional management significantly influences surgical outcomes. Currently, the impact of whey protein supplementation on patients following bariatric surgery remains uncertain. This study aims to explore the role of whey protein supplementation in this patient population through a meta-analytical approach.

**Methods:**

This study was conducted in accordance with the Preferred Reporting Items for Systematic Reviews and Meta-Analyses (PRISMA) 2020 statement. Randomized controlled trials (RCTs) investigating whey protein supplementation in patients after bariatric surgery and published before August 2025 were systematically identified through online databases, including PubMed, the Cochrane Library, and Embase, as well as grey literature sources, including OpenGrey and ProQuest Theses and Dissertations. Data related to anthropometry, body composition, and lipid profiles, including endpoint values and change values, were extracted for quantitative analysis. The quality of evidence was evaluated using the Grading of Recommendations Assessment Development and Evaluation system.

**Results:**

Five RCTs encompassing 267 patients were included in the analysis. The results of the meta-analysis concerning anthropometry and body composition suggested that whey protein supplementation may alleviate the reduction in body weight (*p* = 0.017) and body mass index (*p* = 0.027), which may be attributable to decreases in fat mass (*p* = 0.024) and the preservation of fat-free mass (*p* = 0.003). Conversely, the pooled estimation results suggest that whey protein supplementation may have a limited impact on lipid profiles. The evidence quality for all parameters was rated as moderate to very low.

**Conclusion:**

Current evidence suggests that supplementation with whey protein post-bariatric surgery may offer potential benefits. However, the current evidence remains limited and further studies are needed to confirm our findings.

**Systematic review registration:**

https://www.crd.york.ac.uk/PROSPERO/view/CRD420251126055, identifier (CRD420251126055).

## Introduction

Obesity represents a multifaceted global health challenge characterized by an abnormal accumulation of fat resulting from an energy imbalance (1). Weight gain is attributable to a myriad of interconnected factors, including dysregulation of the endocrine and metabolic systems, genetic predisposition, as well as social and environmental influences (2). The majority of mortality rates associated with obesity are linked to an elevated body mass index (BMI) and are primarily due to cardiovascular diseases (3). Since the early 1980s, more than two billion individuals worldwide have been affected, and this number continues to rise significantly (4, 5). It is projected that by 2030, nearly half of the adult population globally will be classified as overweight or obese (6). Consequently, the treatment and effective management of obesity are critical to addressing global health outcomes (7).

Bariatric surgery is currently regarded as one of the most effective treatment modalities for patients suffering from severe obesity (8). Surgical interventions can facilitate long-term weight reduction in individuals with severe obesity and ameliorate obesity-related comorbidities, potentially reducing associated mortality risk (9, 10). In addition, given that a majority of surgical patients exhibit nutritional and metabolic imbalances, there is an increasing recognition of the importance of nutritional management post-operatively. This is particularly crucial for optimizing surgical outcomes, especially in maintaining long-term weight control and metabolic balance (11, 12). Modern obesity management goes beyond simple weight reduction, focusing on the optimization of body composition by targeting the reduction of fat mass (FM) while simultaneously promoting the increase of lean body mass (LBM) (13). Bariatric surgery can result in substantial reductions in both FM and fat-free mass (FFM), in some cases exceeding the established warning threshold for the proportion of FFM relative to body weight. Appropriate postoperative interventions, such as protein supplementation, may help preserve FFM and thereby optimize body composition (14). Accordingly, adequate protein supplementation, particularly the use of high-quality protein sources, may play a critical role in improving postoperative clinical outcomes (15). However, there remains substantial controversy regarding the appropriate protein supplementation post-surgery.

Variations in protein composition and amino acid profiles may potentially influence clinical outcomes in patients after bariatric surgery (16). Therefore, identifying the appropriate protein formulation is of considerable clinical importance for the nutritional management of patients undergoing bariatric surgery (17). Whey protein is characterized by its rich content of sulfur-containing amino acids and an advantageous ratio of essential amino acids, which has been shown to promote muscle protein synthesis and modulate lipid metabolism, thereby suggesting its potential contribution to the adjustment of fat mass and lipid profiles (18, 19). This effect may offer benefits for the improvement of body composition following bariatric surgery. Furthermore, recent clinical studies have demonstrated that whey protein supplementation can enhance the anthropometric measurements and body composition of patients after bariatric procedures (20, 21). Therefore, postoperative whey protein supplementation may improve clinical outcomes by optimizing body composition, which is consistent with the current therapeutic goals in the clinical management of obesity. In contrast, some perspectives argue that such supplementation may have limited efficacy in postoperative improvements (22). Therefore, there remains a debate on the necessity of incorporating whey protein into the nutritional management of post-bariatric surgery patients. In light of this, we aim to quantitatively compare anthropometry, body composition, and lipid profiles through a meta-analysis to elucidate the role and significance of whey protein supplementation in the post-surgical setting.

## Methods

### Literature retrieval and acquisition

This study has been pre-registered on the PROSPERO website with ID CRD420251126055 and conducted in accordance with the Preferred Reporting Items for Systematic Reviews and Meta-Analyses (PRISMA) 2020 statement (23). The literature search was performed through electronic retrieval of PubMed, the Cochrane Library, and Embase, and was supplemented by a search of grey literature sources, including OpenGrey and ProQuest Theses & Dissertations. Relevant MeSH terms, such as *bariatric surgery*, *body weight*, *body composition*, *lipid profile*, *randomized controlled trial* and so on, were searched both individually and in combination across the different databases, followed by a comparative assessment of the retrieved records. Studies were then progressively screened according to the results generated by the sequential combination of search terms (an example of the PubMed search strategy is provided in Supplementary Table S1). For grey literature, searches were conducted primarily in accordance with the retrieval rules of each database, using either individual terms or combined terms, while maintaining a search framework consistent with that used for the bibliographic databases. No language restrictions were applied to the included publications, although non-English studies were required to provide a matched English title and abstract. In addition, the publication date or study completion date was restricted to before August 2025. Based on this search strategy, all potentially eligible records underwent manual screening by two independent reviewers, and the final decision regarding study inclusion was made by the principle investigator after review of the compiled records. Upon the initial removal of duplicate records across the various databases, further inclusion and exclusion criteria were applied based on the titles, abstracts, and full texts of the articles.

### Inclusion and exclusion criteria

In alignment with the objectives of this study, the inclusion criteria were established as follows: (1) randomized controlled trials (RCTs); (2) supplementation with whey protein as the sole intervention, without limitations regarding the method of administration; (3) studies conducted in the early or late postoperative periods following bariatric surgery, with no specific restrictions on the surgical techniques employed; (4) resistance training was permitted as a background intervention, provided that whey protein supplementation was the only variable differing between the comparison groups; (5) availability of at least one parameter dataset.

The exclusion criteria consisted of: (1) non-RCTs, observational studies, case reports, reviews, and commentaries; (2) studies in which whey protein was administered but the specifics of the intervention were insufficiently detailed; (3) research involving populations not subjected to bariatric surgery; (4) duplicate publications involving the same sample population; and (5) inability to provide any parameter dataset.

### Data extraction and synthesis

Based on the efficacy of whey protein supplementation, we proposed to analyze three key aspects in post-bariatric surgery patients: anthropometry, body composition, and lipid profile analysis. The parameters extracted for anthropometry included body weight and body mass index (BMI), while body composition assessment encompassed fat mass (FM) and fat-free mass (FFM). Lipid profile analysis was derived from blood test results, including total cholesterol (TC), triglycerides (TG), low-density lipoprotein (LDL), and high-density lipoprotein (HDL). We extracted and analyzed endpoint values and change values for these parameters separately. Endpoint values were extracted and compared when baseline measurements were comparable between groups. If baseline values were not inconsistent, endpoint values were not included in the analysis. In addition, change values for the same parameters (if reported) were also extracted and pooled. Accordingly, endpoint values and change values were evaluated separately to provide a more comprehensive assessment of the effects of whey protein following bariatric surgery. Additionally, data collected at various follow-up time points was extracted and subsequently aggregated after stratification based on equivalent follow-up durations.

### Risk of bias assessment and recommendation of evidence levels

All included studies underwent risk assessment for bias using the Cochrane Risk of Bias 2 (24), according to which the bias risks of studies were rated in several domains: bias arising from the randomization process, bias due to deviations from intended interventions, bias due to missing outcome data, bias in measurement of the outcome and bias in selection of the reported result. The summary analysis results for each indicator were evaluated for the quality of evidence using the Grading of Recommendations Assessment, Development and Evaluation (GRADE) system (25). Ultimately, adjustments to the evidence quality rating would depend on a comprehensive evaluation of five downgrading factors and three upgrading factors (26). Quality assessment was performed independently by two investigators. Any discrepancies arising during the assessment process were referred to the principle investigator, who made the final determination.

### Statistical analysis

The data extracted in this study were continuous variables, and the final synthesized analysis was conducted using means and standard deviations for comparison. For the median values accompanied by ranges reported in the original literature, we converted these values into means and standard deviations using appropriate conversion formulas (27, 28). Weighted mean differences (WMDs) with corresponding 95% confidence intervals (CIs) were used to pool continuous outcomes. Heterogeneity was evaluated comprehensively on the basis of clinical heterogeneity, methodological heterogeneity, and the I^2^ statistic. *p* < 0.05 were deemed statistically significant. The STATA software package (version 15.0) was employed for the aggregate calculations, while bias risk assessment was conducted using Review Manager Software (version 5.3). Additionally, the GRADE Profiler (version 3.6) was utilized to evaluate and recommend the quality of evidence.

## Results

### Characteristics of the included studies

**Figure 1:**
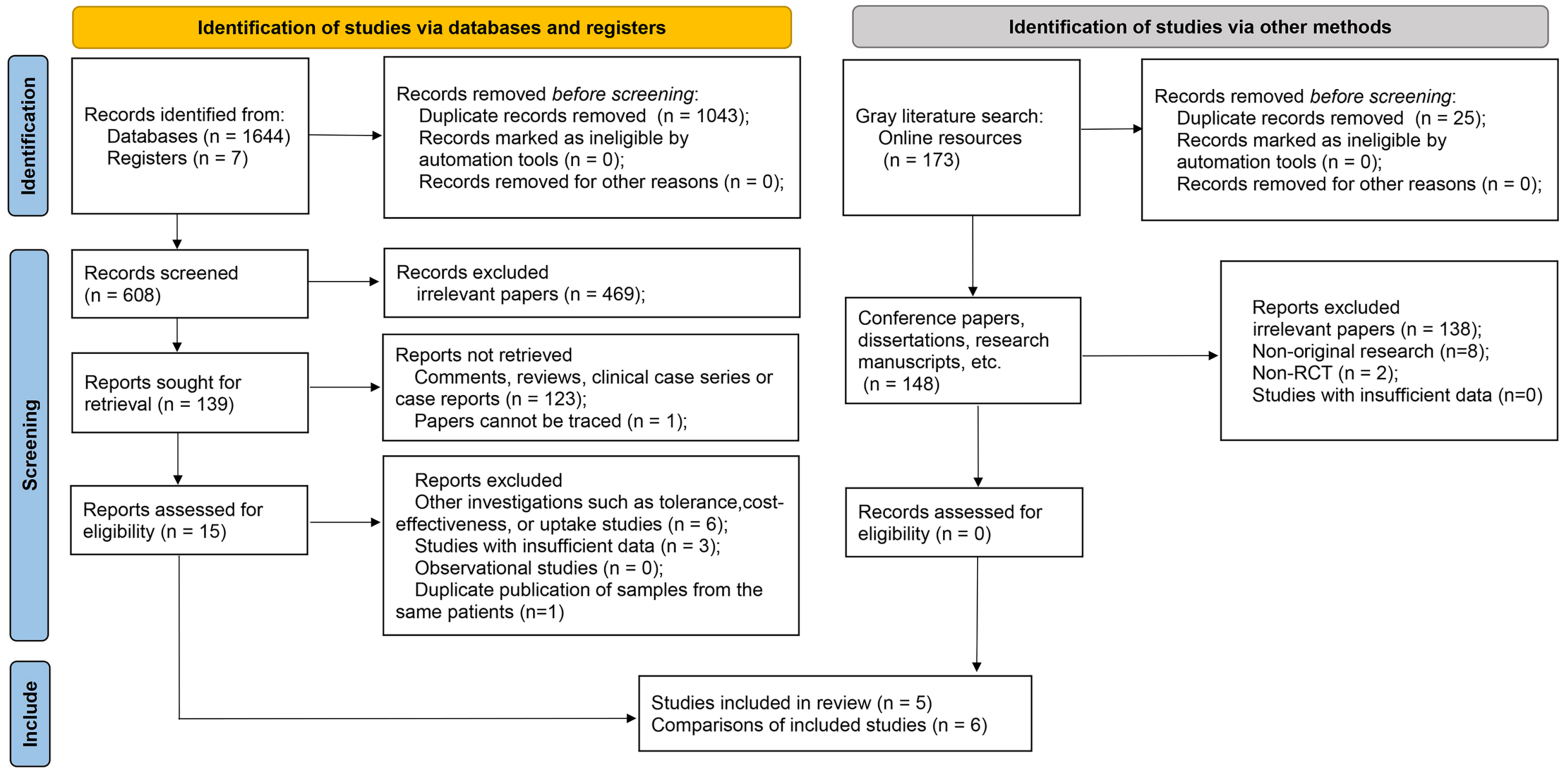
Flow diagram of the process of selecting studies for the current meta-analysis.

Based on relevant search terms and after eliminating duplicate literature, we initially identified 265 pertinent studies. Following a thorough review of titles, abstracts, and full texts, we ultimately selected five RCTs, comprising six distinct comparisons and a total patient sample of 267 for the final quantitative analysis (20–22, 29, 30) (Figure 1). Among these studies, two employed sleeve gastrectomy (SG), two utilized Roux-en-Y gastric bypass (RYGB), and one was based on one anastomosis gastric bypass (OAGB). The follow-up periods across the included studies ranged from 1 month to 6 months, categorized into 5 distinct recording time points: 1 month, 8 weeks, 12 weeks (3 months), 16 weeks, and 6 months. Lamarca et al. (22) and Oppert et al. (30) included 4 arms and 3 arms, respectively, and included samples that involved resistance training. Notably, the 4 groups in Lamarca et al. comprised 2 comparisons, while in the report by Oppert et al., only 2 arms were retained for comparison between whey protein and the control group. Regarding the duration of the postoperative interval, Gomes et al. (20) and Lamarca et al. (22) conducted their trails 2 years after the surgery, whereas the other RCTs commenced their assessments immediately following the surgical procedures. Furthermore, although the intake protocols for whey protein varied among the studies and some RCTs incorporated multiple arms, whey protein was consistently employed as the sole intervention variable for the outcome analyses (Table 1).

**Table 1:** Characteristics of included studies.

Author/Year	Design	Sample size	Surgery type	Study arm (intervention)	Mean age (Con. vs. Exp.)	Baseline BMI (Con. vs. Exp.)	Gender (Female %)	Duration	Completion rate	Doses of whey protein	Available Parameters	Remarks
Gomes 2017 (20)	RCT	30	RYGB	2 arms	49.0 ± 10 vs. 41.0 ± 10	35 ± 4 vs. 36 ± 6	100%	8th/16th weeks	88.23%	0.5 g/kg/day	TC, TG, LDL (endpoint value for all)	—
Gunes 2019 (29)	RCT	60	SG	2 arms	43.52 ± 8.35 vs. 40.28 ± 11.4	45.93 ± 6.52 vs. 46.21 ± 4.97	85%	1st/3rd/6th months	88.23%	1.2 g/kg/day	Body weight (endpoint value), BMI (endpoint value), FM (change value), FFM (change value)	—
Lamarca 2021 (22)	RCT	63	RYGB	4 arms	39.8 ± 7.8 vs. 40.6 ± 10.4 AND 40.0 ± 8.7 vs. 41.0 ± 6.4	29.3 ± 4.4 vs. 30.1 ± 5.7 AND 29.8 ± 6.1 vs. 29.4 ± 5.4	88.89%	12th weeks	52.94%	30 g/day	Body weight, BMI, FM, FFM, TC, TG, LDL, HDL (endpoint value and change value for all)	This RCT contains 4 arms but only 2 comparisons were included. Comparison 1: control vs. whey protein. Comparison 2: control + resistance training vs. whey protein + resistance training.
Oppert 2018 (30)	RCT	47	RYGB	3 arms	43.9 ± 10.7 vs. 42.5 ± 8.7	43.6 ± 6.2 vs. 43.3 ± 6.0	100%	6th months	100%	48 g/day	Body weight, BMI, FM (change value for all)	This RCT contains 3 arms and the group of whey protein + resistance training (11 participants) was excluded in this meta-analysis. Only the comparison of control vs. whey protein was included.
Sabooni 2025 (21)	RCT	78	OAGB	2 arms	40.5 ± 10.7 vs. 41.47 ± 8.65	46.49 ± 5.63 vs. 43.4 ± 2.99	89.74%	1st/3rd months	83.87%	30 g/day	BMI (change value), TC (endpoint value), TG (endpoint value), LDL (endpoint value), HDL (endpoint value)	—

RCT, randomized controlled trial; RYGB, Roux-en-Y gastric bypass; SG, sleeve gastrectomy; OAGB, one anastomosis gastric bypass; BMI, body mass index; FM, fat mass; FFM, fat free mass; TC, total cholesterol; TG, triglyceride; LDL, low density lipoprotein; HDL, high density lipoprotein.

### Assessment of literature quality

**Figure 2:**
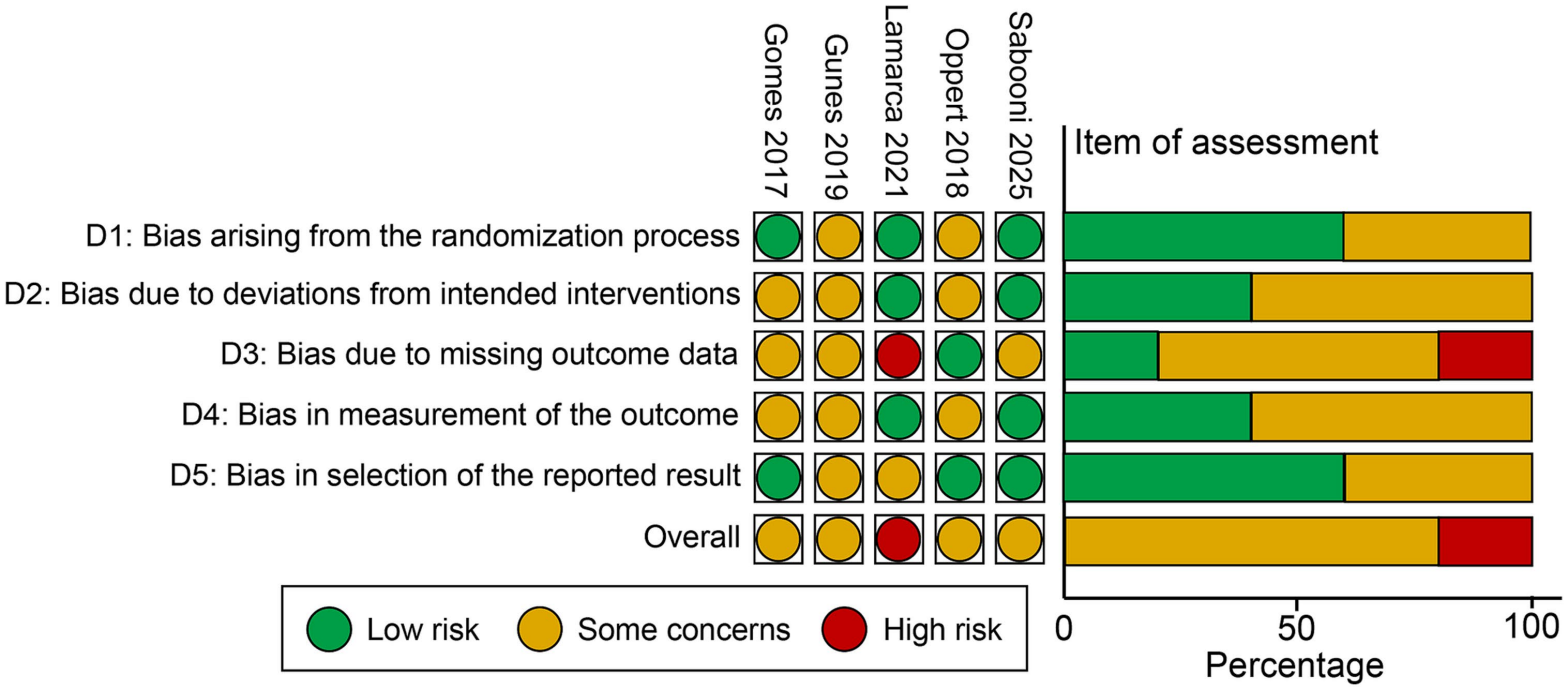
Bias assessment regarding each risk of bias item and summary of the included studies.

All five studies included in this analysis employed random allocation methods, and three explicitly reported allocation concealment. The remaining two did not clearly describe this procedure, which may give rise to some concerns. Two included studies reported the use of a double-blind design, whereas the other three did not clearly specify their blinding procedures, which may have introduced bias related to intended interventions and outcome measurement. One study may have been at risk of bias because of substantial loss to follow-up, and two studies did not clearly state whether selective reporting was present. Overall, the included studies may be subject to a relatively high risk of bias (Figure 2).

### Impact of whey protein on post-operative patients

**Figure 3:**
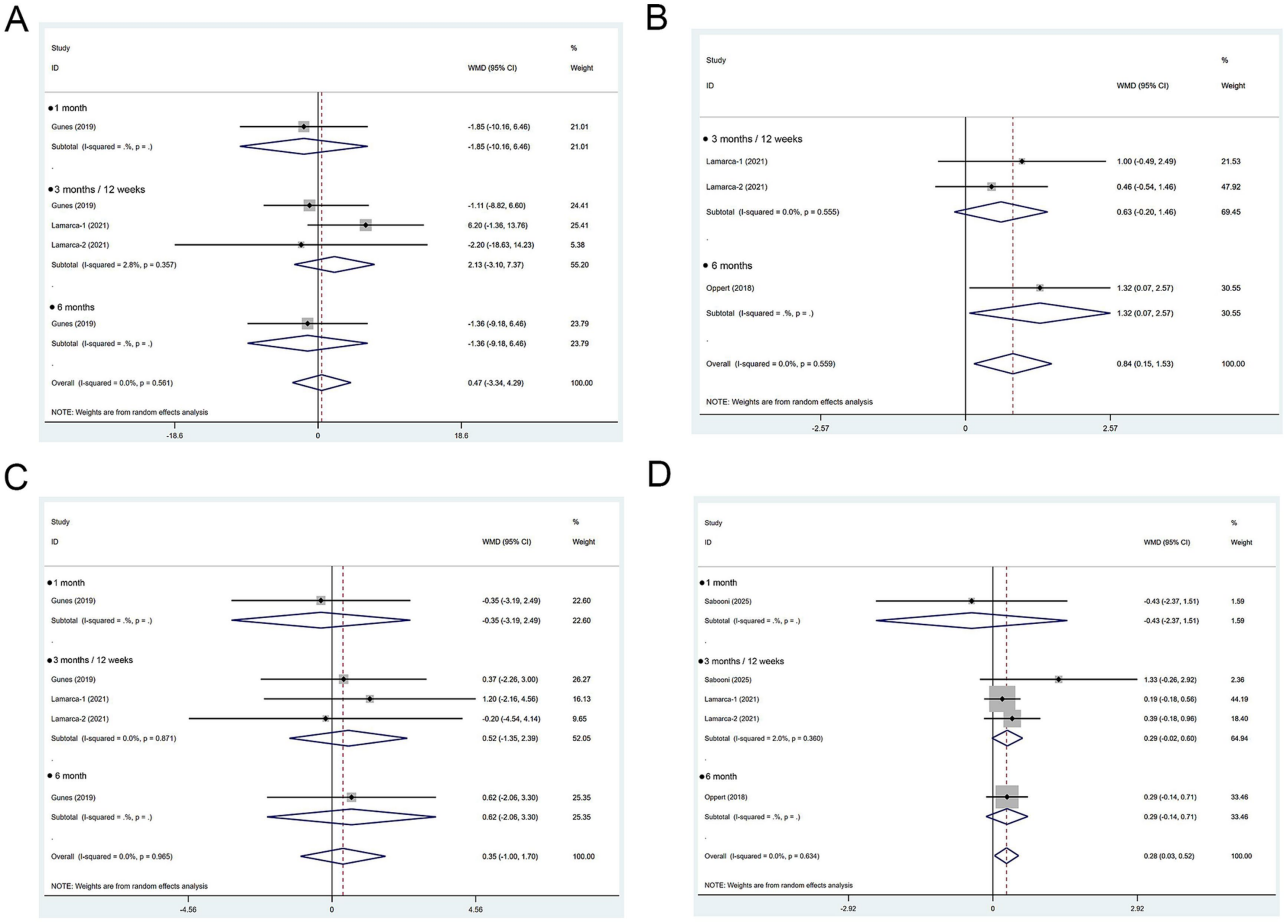
Forest plot comparing the whey protein supplementation group to the control group concerning **(A)** the endpoint value of body weight, **(B)** the change in body weight, **(C)** the endpoint value of body mass index (BMI), and **(D)** the change in BMI.

Given the characteristics of the included studies, we found that there were substantial differences in the clinical managements, background interventions, trial designs, and so on. Therefore, we suggested that the RCTs included in this study exhibit clinical and methodological heterogeneity. Accordingly, a random-effects model was applied for all parameter comparisons. We first conducted a meta-analysis to observe anthropometric changes, specifically focusing on body weight and BMI. Two RCTs reported body weight endpoint values across three different time points. The results revealed no significant differences in body weight outcomes between the two groups [WMD (95% CI) = 0.47 (−3.34, 4.29), *p* = 0.807] (Figure 3A). However, when examining the specific changes (change values) in body weight, the results suggested that whey protein may mitigate weight loss [WMD (95% CI) = 0.84 (0.15, 1.53), *p* = 0.017] (Figure 3B). Similarly, meta-analysis for BMI indicated no difference in the BMI endpoint values between whey protein consumers and the control group [WMD (95% CI) = 0.35 (−1.00, 1.70), *p* = 0.611] (Figure 3C). Nonetheless, the change in BMI for patients supplementing with whey protein was indicated to be less than that observed in the control group [WMD (95% CI) = 0.28 (0.03, 0.52), *p* = 0.027] (Figure 3D), which is consistent with the body weight change findings.

**Figure 4:**
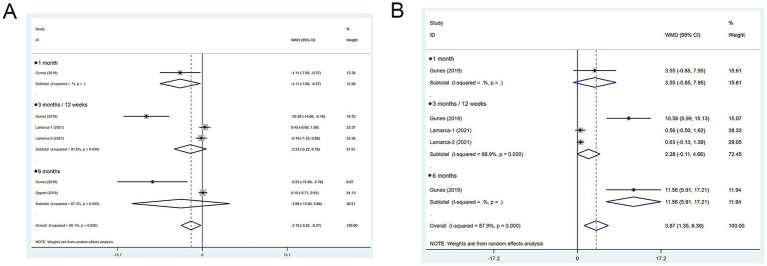
Forest plot comparing the whey protein supplementation group to the control group with respect to the change in **(A)** fat mass and **(B)** fat-free mass.

For the assessment of body composition, due to the availability of only one study reporting endpoint values for these parameters, pooled estimation was not conducted. However, regarding changes in FM, a quantitative synthesis of three RCTs indicated that supplementation with whey protein may enhance changes in FM, thereby contributing to a reduction in fat mass [WMD (95% CI) = −2.10 (−3.92, −0.27), *p* = 0.024] (Figure 4A). In contrast, the quantitative pooled estimation analysis for FFM suggested that whey protein intake may increase the positive change in fat-free mass, resulting in an elevation of FFM levels [WMD (95% CI) = 3.87 (1.35, 6.39), *p* = 0.003] (Figure 4B).

**Figure 5:**
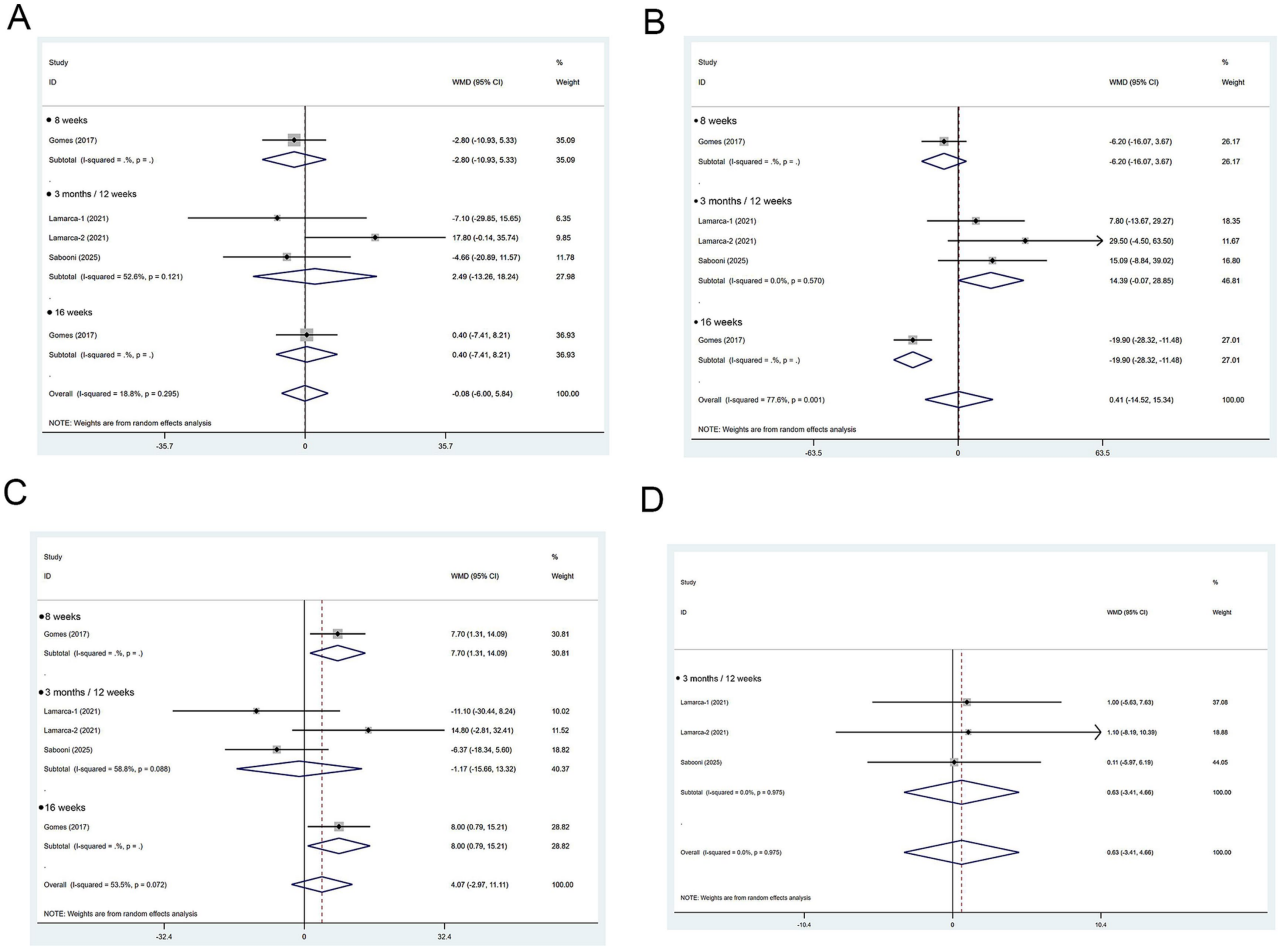
Forest plot comparing the whey protein supplementation group to the control group regarding the endpoint values of **(A)** total cholesterol, **(B)** triglycerides, **(C)** low-density lipoprotein (LDL), and **(D)** high-density lipoprotein (HDL).

To assess the influence of whey protein intake on lipid profiles, we extracted and aggregated blood test data pertaining to TC, TG, LDL and HDL. As there was only one study reporting changes in these metrics, we performed a pooled analysis of the relevant endpoint values. The quantitative analysis indicated that supplementation with whey protein in post-operative patients did not appear to affect TC [WMD (95% CI) = −0.08 (−6.00, 5.84), *p* = 0.974] or TG levels [WMD (95% CI) = 0.41 (14.52, 15.34), *p* = 0.957] (Figures 5A,B). Similarly, whey protein supplementation appeared to have no significant effect on LDL [WMD (95% CI) = 4.07 (−2.97, 11.11), *p* = 0.257] or HDL levels [WMD (95% CI) = 0.63 (−3.41, 4.66), *p* = 0.761] (Figures 5C,D).

### Potential publication bias and recommendation of evidence

Our quality assessment suggested that the included studies may be affected by high risk of bias. Furthermore, several characteristics of the included studies, such as differences in whey protein supplementation protocols, variations in surgical techniques, the inclusion of participants who engaged in resistance training, and differences in postoperative assessment time points, may have also exerted a potential influence on the results. Given the limited number of included studies, the risk of publication bias may not be meaningfully assessed in the present study. Therefore, we applied the GRADE system to evaluate the overall certainty of the evidence for the above outcomes. Following an evaluation based on different parameter criteria, our research does not present high-quality evidence, with all evidence levels categorized as ranging from moderate to very low (Table 2), indicating that the overall evidence remains limited.

**Table 2:** The quality and recommendation of the evidence according to the GRADE system.

**Outcomes**	**Effect size (95% CI)**		**Absolute effect**	**Significant**	**No. of participants** **(studies)**	**Quality of the evidence** (GRADE)*
	**Control arm**	**Whey protein arm**				
BW (outcome value)	92.13 (79.49 to 104.78)	92.18 (82.77 to 102.58)	0.47 (−3.34, 4.29)	No	123 (2 RCTs)	⊕⊕ Low^1, 3^
BW (value of changes)	−8.82 (−30.08 to 12.44)	−7.89 (−24.47 to 8.69)	0.84 (0.15, 1.53)	Yes	99 (2 RCTs)	⊕ ⊕ ⊕ Moderate^3^
BMI (outcome value)	34.37 (30.04 to 38.71)	34.73 (30.79 to 38.66)	0.35 (−1.00, 1.70)	No	123 (2 RCTs)	⊕⊕ Low^1, 3^
BMI (value of changes)	−8.45 (−15.20 to −1.70)	−8.08 (−13.77 to −2.39)	0.28 (0.03, 0.52)	Yes	167 (3 RCTs)	⊕ ⊕ ⊕ Moderate^3^
FM (value of changes)	−11.68 (−22.09 to −1.27)	−15.56 (−25.39 to −5.73)	−2.10 (−3.92, −0.27)	Yes	159 (3 RCTs)	⊕⊕ Low^2, 3^
FFM (value of changes)	−2.16 (−4.09 to −0.24)	1.51 (0.35 to 2.66)	3.87 (1.35, 6.39)	Yes	123 (2 RCTs)	⊕⊕ Low^2, 3^
TC (outcome value)	163.68 (157.61 to 169.75)	163.74 (159.26 to 168.22)	−0.08 (−6.00, 5.84)	No	160 (3 RCTs)	⊕⊕ Low^1, 3^
TG (outcome value)	97.24 (78.37 to 116.11)	100.37 (88.09 to 112.66)	0.41 (14.52, 15.34)	No	160 (3 RCTs)	⊕ Very low^1, 2, 3^
LDL (outcome value)	86.41 (81.81 to 91.00)	91.13 (88.74 to 93.52)	4.07 (−2.97, 11.11)	No	160 (3 RCTs)	⊕ Very low^1, 2, 3^
HDL (outcome value)	55.25 (44.95 to 65.55)	55.94 (43.87 to 68.01)	0.63 (−3.41, 4.66)	No	130 (2 RCTs)	⊕⊕ Low^1, 3^

*GRADE Working Group grades of evidence: High quality (⊕ ⊕ ⊕⊕): Further research is very unlikely to change our confidence in the estimate of effect. Moderate quality (⊕ ⊕ ⊕): Further research is likely to have an important impact on our confidence in the estimate of effect and may change the estimate. Low quality (⊕⊕): Further research is very likely to have an important impact on our confidence in the estimate of effect and is likely to change the estimate. Very low quality (⊕): We are very uncertain about the estimate. 1. The confidence intervals overlapped the line of no effect (−1); 2. High statistical heterogeneity (*I*^2^ > 50%) (−1). 3. High or unclear risk of bias (−1).

### Subgroup analysis

In light of the evident confounding factors across the included studies, subgroup analyses were performed according to surgical procedure, postoperative interval, and the use of resistance training. Whey protein supplementation may help prevent excessive weight loss in patients undergoing SYGB, whereas its effect on preserving fat-free mass may be more pronounced in those undergoing SG. With respect to the timing of supplementation, initiating whey protein immediately after surgery may be beneficial for maintaining fat-free mass, whereas supplementation introduced more than 2 years postoperatively may provide no clinically meaningful benefit. Finally, subgroup analyses based on whether patients engaged in resistance training suggested that whey protein supplementation may be beneficial only for those who did not perform resistance training, while it may not confer additional clinical benefit in patients who did (Supplementary Table S2).

## Discussion

Our findings suggest that whey protein supplementation may mitigate reductions in body weight and BMI. Furthermore, statistical analysis of changes in FM and FFM indicates that whey protein supplementation may reduce FM while preserving FFM. Although reduced weight loss following bariatric surgery might be undesirable, rapid postoperative weight reduction, particularly when accompanied by a loss of fat-free mass, may adversely affect surgical outcomes. Conversely, preserving FFM to achieve a more favorable body composition may better align with postoperative goals. The reduction in FM combined with the preservation of FFM may be attributed to whey protein’s ability to promote muscle protein synthesis, as well as its potential role in facilitating the *β*-oxidation of fatty tissue (31). Therefore, whey protein supplementation may enhance postoperative clinical benefit by optimizing body composition through reducing FM and preserving FFM.

The results of blood lipid profile analyses indicate that the effects of whey protein on adipose tissue are not well-represented in lipid metabolism assessments. Whey protein may exert its primary effects by stimulating skeletal muscle protein synthesis and enhancing myofibrillar protein synthesis rates (32, 33). This demand may be pronounced in patients after bariatric surgery. Consequently, ingested whey protein may be preferentially utilized for muscle protein accretion, whereas its regulatory effects on fat metabolism may be insufficient to produce detectable changes in lipid profile measurements. In addition, given the clinical heterogeneity among the included studies, these confounding factors may have partially obscured the effects of whey protein on the blood lipid profile.

Our study systematically evaluated the role of whey protein in patients post-bariatric surgery from multiple perspectives, providing evidence that whey protein may contribute to the maintenance of body weight and BMI by promoting FFM preservation and reducing FM, which is congruent with its molecular mechanisms of action in the body. Prior to our research, only one systematic review had investigated the effects of protein supplementation on lean body mass following bariatric surgery (34). This previous study included various types of protein but ultimately did not reach definitive conclusions, emphasizing a lack of qualitative and quantitative studies focusing on specific protein types. In contrast, our study is the first to quantitatively assess the impact of whey protein supplementation on post-bariatric surgery patients, arriving at preliminary conclusions that indicate its beneficial effects.

In the current study, among the six comparisons included, one comparison from Lamarca et al. involved subjects engaged in resistance training. Postoperative exercise training has been shown to enhance the outcomes following bariatric surgery, particularly in terms of body composition modulation (35). However, regarding the comparison between resistance training and whey protein supplementation, only two of the included RCTs reported relevant findings, both of which asserted that there was no significant difference between the two interventions in improving postoperative outcomes. Nevertheless, their combined application may yield synergistic effects (22, 30). The subgroup analyses suggested that whey protein supplementation did not appear to provide additional benefit in patients who engaged in postoperative resistance training. Its potential benefits were mainly observed in patients who did not perform resistance training, particularly with respect to improvements in body composition. This finding may indicate that resistance training alone is sufficient to improve body composition in patients after bariatric surgery, thereby reducing the incremental benefit of whey protein supplementation. It also suggests that the inclusion of patients undergoing resistance training may have influenced the overall results of this study.

Two of the included RCTs commenced 2 or more years postoperatively (20, 22), while the other RCTs were initiated immediately following surgery. Although bariatric surgery has demonstrated its efficacy in addressing severe obesity, it may also present challenges in the long term, such as varying degrees of recurrent weight gain and nutritional deficiencies. These issues may lead to differences in the basal metabolic rates among the different cohorts (36). The subgroup analyses also indicated that whey protein supplementation may be effective only when initiated immediately after bariatric surgery, whereas no clear benefit was observed in patients who began supplementation more than 2 years postoperatively. This finding suggests the presence of a potential therapeutic window for postoperative whey protein supplementation, which may be related to the re-establishment of long-term basal metabolic balance after surgery. Consequently, while there is currently no available literature directly linking the effects of postoperative protein supplementation to the duration of time since surgery, we hypothesize that variations in postoperative time may represent an important confounding factor affecting the results.

In terms of surgical techniques, the included RCTs encompassed 3 distinct bariatric procedures: SG, RYGB, and OAGB. Among these, SG is the most widely utilized technique, celebrated for its weight loss efficacy and safety profile. However, its short-term effectiveness in decreasing fat mass and its long-term ability to preserve lean body mass appear to be inferior to those of RYGB (37). Furthermore, as an evolution of RYGB, OAGB demonstrates comparable efficacy in treating severe obesity (38). While its safety profile remains to be fully established, its therapeutic effects may even surpass those of SG (39). In addition, the benefits of whey protein supplementation appeared to differ according to the type of bariatric procedure performed. In particular, differences in nutrient absorption between SYGB and SG may influence the absorption and utilization of whey protein. As a result, SYGB may be associated with greater long-term weight stability, whereas SG may be more favorable for maintaining nutrient absorption and thereby allowing whey protein to exert a more pronounced effect on body composition (40). Therefore, although each surgical method exhibits commendable effectiveness in the management of severe obesity, their respective outcomes are not entirely analogous, which may introduce new potential clinical heterogeneity into the aggregated analysis.

Regarding the protein supplementation protocols, the doses of whey protein utilized in the included RCTs varied, and differing protein dosages may lead to differential effects on outcomes following bariatric surgery (41). Specifically, for whey protein, whether used in conjunction with resistance training or not, its role in promoting muscle protein synthesis exhibits a dose-dependent effect (33, 42), which may directly influence the comparative results pertaining to body composition. Therefore, although no existing studies have verified whether the effect of whey protein supplementation applies to post-bariatric surgery patients, we hypothesize that these variabilities may still introduce potential bias into the study findings.

It should also be noted that although the subgroup analyses suggested that the benefits of postoperative whey protein supplementation may be associated with surgical procedure, postoperative interval, and resistance training status, the limited number of included studies restricts the strength of these inferences. In addition, the classification of certain subgroups was potentially overlapping. For example, Lamarca et al. (22) was the only study that included resistance training, and it was also a study in which whey protein supplementation was initiated 2 years after surgery. Because the subgroup analyses indicated no apparent benefit in either patients more than 2 years postoperatively or those undergoing resistance training, it remains unclear whether this finding was attributable to postoperative interval, resistance training, or both. Further studies are needed to clarify this issue.

Considering the aforementioned factors, while whey protein supplementation was designated as the sole intervention across all comparisons, the existence of multiple confounding variables and the unclear magnitude of their impact on outcomes necessitates acknowledgment of potential bias in the present study. Furthermore, in some comparisons of continuous outcomes, the pooled estimates were based on data converted from medians rather than derived from original datasets. Together with the overall small sample size, this may have affected the robustness of our findings.

## Conclusion

In summary, our study provides preliminary evidence that whey protein supplementation may offer potential benefits for patients after bariatric surgery. Whey protein supplementation may help preserve fat-free mass after bariatric surgery; however, current evidence remains limited by small sample sizes, methodological heterogeneity, and moderate to very low certainty evidence. Therefore, our conclusions require further validation through additional high-quality, large-scale RCTs.

## Data Availability

The original contributions presented in the study are included in the article/Supplementary material, further inquiries can be directed to the corresponding author/s.
