# Supplementary material for: The potential effect of whey protein supplementation in post-bariatric surgery patients: a systematic review and meta-analysis of randomized controlled trials
Source: Front Nutr. 2026 Jun 23;13:1832335. doi: 10.3389/fnut.2026.1832335 (PMC13337453; doi:10.3389/fnut.2026.1832335)
Supplement: Supplementary file 1 [file Data_Sheet_1.PDF]

Supplementary Tables

Table S1. Example of search order in Pubmed.

Order	MeSH
1#	bariatric surgery
2#	sleeve gastrectomy
3#	one anastomosis gastric bypass
4#	Roux-en-Y gastric bypass
5#	duodeno-ileal bypass
6#	duodenal switch
7#	transit bipartition
8#	1# OR 2# OR 3# OR 4# OR 5# OR 6# OR 7#
9#	body weight
10#	body mass index
11#	fat mass
12#	fat free mass
13#	cholesterol
14#	triglyceride
15#	lipoprotein
16#	anthropometry
17#	body composition
18#	lipid profile
19#	9# OR 10# OR 11# OR 12# OR 13# OR 14# OR 15# OR 16# OR 17# OR 18#
20#	whey
21#	randomized
22#	randomized controlled trial
23#	21# OR 22#
24#	8# AND 19# AND 20# AND 23#
Search strings	((((((((bariatric surgery) OR (sleeve gastrectomy)) OR (one anastomosis gastric bypass)) OR (Roux-en-Y gastric bypass)) OR (duodeno-ileal bypass)) OR (duodenal switch)) OR (transit bipartition)) AND ((((((((((body weight) OR (body mass index)) OR (fat mass)) OR (fat free mass)) OR (cholesterol)) OR (triglyceride)) OR (lipoprotein)) OR (anthropometry)) OR (body composition)) OR (lipid profile))) AND (whey)) AND ((randomized) OR (randomized controlled trial))

Table S2. Subgroup analysis regarding surgical type, post-operative period and resistence training

Subgroup			Surgical type		Post-operative period		With resistence training	
Item			RYGB	The others	Perioperative	over 2 years	No	Yes
Anthropometry	body weight (endpoint value)	No. of comparisons	2	1	1	2	2	1
		WMD (95% CI)	4.73 (-2.14, 11.60)	-1.42 (-6.00, 3.16)	-1.42 (-6.00, 3.16)	4.73 (-2.14, 11.60)	0.63 (-3.29, 4.54)	-2.20 (-18.63, 14.23)
		P	0.181	0.542	0.542	0.181	0.751	0.794
	body weight (change value)	No. of comparisons	3	0	1	2	2	1
		WMD (95% CI)	0.84 (0.15, 1.53)	—	1.32 (0.07, 2.57)	0.63 (-0.20, 1.46)	1.19 (0.23, 2.14)	0.46 (-0.54, 1.46)
		P	0.017	—	0.042	0.146	0.022	0.368
	body mass index (endpoint value)	No. of comparisons	2	1	1	2	2	1
		WMD (95% CI)	0.68 (-1.98, 3.33)	0.24 (-1.33, 1.80)	0.24 (-1.33, 1.80)	0.68 (-1.98, 3.33)	0.41 (-1.01, 1.83)	-0.20 (-4.54, 4.14)
		P	0.629	0.779	0.779	0.629	0.577	0.933
	body mass index (change value)	No. of comparisons	3	1	2	2	3	1
		WMD (95% CI)	0.26 (0.01, 0.51)	0.54 (-1.18, 2.26)	0.34 (-0.16, 0.84)	0.25 (-0.06, 0.56)	0.25 (-0.02, 0.52)	0.39 (-0.18, 0.96)
		P	0.040	0.544	0.197	0.116	0.075	0.183
Body composition	fat mass (change value)	No. of comparisons	3	1	2	2	3	1
		WMD (95% CI)	0.09 (-0.46, 0.64)	-7.64 (-11.98, -3.31)	-5.52 (-11.11, -0.07)	0.08 (-0.66, 0.82)	-3.25 (-5.83, -0.67)	-0.19 (-1.24, 0.86)
		P	0.757	<0.001	0.053	0.838	0.014	0.728
	fat-free mass (change value)	No. of comparisons	2	1	1	2	2	1
		WMD (95% CI)	0.61 (-0.01, 1.22)	8.39 (3.29, 13.49)	8.39 (3.29, 13.49)	0.61 (-0.01, 1.22)	6.22 (0.40, 12.03)	0.63 (-0.13, 1.39)
		P	0.054	0.001	0.001	0.054	0.043	0.102
Lipid profiles	total cholesterol (endpoint value)	No. of comparisons	3	1	1	3	3	1
		WMD (95% CI)	0.83 (-6.41, 8.08)	-4.66 (-20.89, 11.57)	-4.66 (-20.89, 11.57)	0.83 (-6.41, 8.08)	-1.81 (-6.99, 3.38)	17.80 (-0.14, 35.74)
		P	0.830	0.574	0.574	0.830	0.495	0.059
	triglycerides (endpoint value)	No. of comparisons	3	1	1	3	3	1
		WMD (95% CI)	-2.96 (-18.50, 12.58)	15.08 (-8.84, 39.02)	15.08 (-8.84, 39.02)	-2.96 (-18.50, 12.58)	-4.02 (-18.22, 10.19)	29.50 (-4.50, 63.50)
		P	0.719	0.219	0.219	0.719	0.586	0.115
	low-density lipoprotein (endpoint value)	No. of comparisons	3	1	1	3	3	1
		WMD (95% CI)	6.93 (0.97, 12.88)	-6.37 (-18.34, 5.60)	-6.37 (-18.34, 5.60)	6.93 (0.97, 12.88)	2.56 (-5.13, 10.25)	14.80 (-2.81, 32.41)
		P	0.017	0.298	0.298	0.017	0.537	0.113
	high-density lipoprotein (endpoint value)	No. of comparisons	2	1	1	2	2	1
		WMD (95% CI)	1.03 (-4.36, 6.43)	0.11 (-5.97, 6.19)	0.11 (-5.97, 6.19)	1.03 (-4.36, 6.43)	0.52 (-3.96, 5.00)	1.10 (-8.19, 10.39)
		P	0.709	0.972	0.972	0.709	0.825	0.818
